# Three-dimensional imaging and quantitative analysis in CLARITY processed breast cancer tissues

**DOI:** 10.1038/s41598-019-41957-w

**Published:** 2019-04-04

**Authors:** Yi Chen, Qi Shen, Sharla L. White, Yesim Gokmen-Polar, Sunil Badve, Laurie J. Goodman

**Affiliations:** 1 ClearLight Biotechnologies, LLC (formerly known as ClearLight Diagnostics, LLC), 428 Oakmead Pkwy, Sunnyvale, CA 94085 USA; 2 Department of Pathology and Laboratory Medicine, Indiana University School of Medicine, Indianapolis, IN 46202 USA

## Abstract

The tumor microenvironment can be spatially heterogenous, which makes it challenging to fully characterize with standard 2D histology-based methods. In this study, we determined the feasibility of a CLARITY tissue-processing approach to analyze biopsies from breast cancer patients. Formalin-fixed human breast cancer core-needle biopsy specimens, were embedded, lipid-cleared, and multiplexed immunostained to identify key biomarkers (pan-cytokeratin, Ki67, CD3). Confocal microscopy was then used to image the specimens after refractive index matching. These data sets were then quantitatively compared to conventional slide-based FFPE histology. Using CLARITY, the gross and cellular morphology of the tissues were well preserved, and high optical transparency was achieved, with the exception of fibrotic regions. Specific staining of various cellular and nuclear markers was achieved using optimized antibody conditions. Manually determined composite Ki67 scores from the CLARITY datasets agreed with histology results. However, the CLARITY datasets (3D) revealed variation in the intra-tumoral Ki67 expression that was not evident in individual FFPE sections (2D). We further demonstrated that archived FFPE clinical specimens can be CLARITY-processed, immunostained, and imaged. In short, CLARITY-processed specimens may enable a more accurate, unbiased analysis of tumor samples in comparison to conventional slide-based histology, thus allowing for improved visualization of intra-tumoral heterogeneity.

## Introduction

In the area of tumor biology, technologies are lacking to merge cellular phenotypic information with three-dimensional (3D) spatial analysis of tissues. There remains a need for a quantitative multiplexed analysis of key biomarkers in cancer specimens, whereby complex spatial patterns of cells, as well as tissue architecture can be reproducibly measured in 3D. It has become clear that the heterogeneity within the tumor microenvironment (TME) contributes significantly to the development and eventual metastasis of cancer, as well as the response and resistance to treatment^1^. Current technologies utilized for preclinical, diagnostic, prognostic, and predictive clinical cancer research are dependent upon two-dimensional (2D) analysis of formalin-fixed paraffin embedded (FFPE) tissue sections (5–10 µm). Other technologies, that utilize flow cytometry, real-time polymerase chain reaction, or next generation sequencing to analyze tumors, lack the ability to correlate key quantitative information while maintaining the architecture and morphology of the TME^2^.

Recent publications, from small studies, have demonstrated key spatial relationships between T-cell phenotypes and key tumor biomarkers in the TME that show prognostic and/or predictive clinical outcomes^3^. Unfortunately, these techniques suffer from small sampling of the tissue when derived from 5-micron thin sections. However, a 3D volumetric assessment of tumors, even in the case of small tissue sampling, may increase the likelihood to determine statistically significant patterns generated by biological processes, as compared to current 2D microscopy methods. Thus, the ability to analyze intact tissues may facilitate the identification of clinically relevant features. Some possible examples, would be a new avenue of pathological grading criteria of larger samples or identifying key spatial relationships within the TME, potentially yielding powerful predictions that extend beyond simple abundance of key biomarkers^1^.

Furthermore, the ability to observe molecular and structural heterogeneity in a dynamic neoplasm, such as cancer, can be crucial for accurate diagnosis, treatment, and predicting recurrence. Understanding the intricacies of both inter-tumor and intra-tumor heterogeneity have become important goals since they have implications on identifying reliable prognostic and predictive molecular biomarkers in clinical oncology^4–7^. In particular, breast cancers are known to exhibit a high level of intra-tumor variability for standard biomarkers such as estrogen receptor (ER), progesterone receptor (PR), human epidermal growth factor receptor 2 (HER2), epidermal growth factor receptor (EGFR), and the proliferative marker Ki67, which can result in divergent outcomes^7^.

The last ten years has seen a surge in methods and reagents to aid in whole tissue processing and subsequent 3D imaging. CLARITY is one novel approach that is innovative in both function and utility, and has been applied broadly to the field of neurobiology, primarily as a qualitative tool. The technology enables the formation of a hydrogel matrix (HM) by crosslinking biological molecules to a 3D network of hydrophilic polymers, followed by lipid clearing to generate a transparent and structurally intact tissue. The tissue then can be labeled with macromolecules and imaged without destruction of the tissue^8–12^. Other tissue clearing methods can generally be classified as either an aqueous/hyperhydration (Scale, CUBIC), organic solvent-based (3DISCO, iDISCO, uDISCO, BABB, DMSO), or a refractive index (RI) immersion matching (Ce3D, SeeDB, ClearT)^13,14^. However, the CLARITY technique results in maintenance of tissue structural integrity, allowing multiple rounds of staining and de-staining, is compatible with endogenous fluorescence and long-term tissue storage, and has been shown to be compatible with both active and passive clearing methods^8,10,15^.

Although the CLARITY technique was originally conceived and applied to the field of neuroscience^15–19^, its usage has extended to other organs^10,20–24^. Several other groups have modified parameters that affect clearing and antibody penetration, such as temperature^9,10^, HM composition^9,10,22,24,25^, and clearing reagents^10,24,25^. Yet, the majority of these studies have been performed in non-cancerous tissues with unoptimized antibody conditions, which may contribute to insufficient molecular probe penetration resulting in a “sandwich” like staining pattern observed in thick tissue^10^. Furthermore, protein loss associated with lipid clearing is another important unknown factor. Previous publications have noted minimal total protein loss by using BCA protein quantification^8,9,24^. However, the matter of specific epitope preservation post-processing remains inconclusive.

It is unknown if the CLARITY method could be properly controlled or standardized for use with a TME relevant multiplexed panel of biomarkers in human cancer specimens; however, results from both our lab and others have shown early feasibility to perform singleplex, as well as multiplex immunostaining in fresh, frozen, formalin-fixed pre-clinical mouse and human cancer tissues (breast, pancreas, non-small cell lung cancer (NSCLC), lymph node) by the CLARITY method^10,21,26^. Yet, the more applicable question of cross-validation to gold standard histopathology techniques remains, and if archived FFPE blocks can be processed successfully through the CLARITY technique and yield comparable results to standard 2D thin-section methods.

In this paper we demonstrate the feasibility of multiplex fluorescent immunoassay staining, with an optimized antibody panel, in CLARITY processed tissues. The multiplex panel comprised of three antibodies: pan-cytokeratin (pan-CK), Ki67, and CD3 (representative of the cytoplasmic, nuclear, and membrane compartments, respectively) was selected. Pan-CK, an abundant and highly-expressed cytoplasmic protein homogenously expressed in xenograft tumors, was used as an optimal antigen for preliminary antibody titrations and subsequent breast cancer tissue staining. We chose the proliferative marker, Ki67, to represent the nuclear compartment, and to serve as a representative marker for manual counting to compare the quantitative analysis of 3D images to standard 2D method. Ki67 has been found to display variable expression with two prominent types of intra-tumoral heterogeneity: an increased tumor edge staining gradient or prevalent Ki67 staining, “hot spots”^27,28^. While multiple sampling and scoring from the different locations of the tissue exists, there is no consensus in methodology for scoring samples across different studies^28,29^. As such, a nondestructive method might be considered a more accurate assessment for markers like Ki67. CD3 is a critical T cell membrane marker and has been frequently studied in the immuno-oncology field, and it broadly can measure the tumor infiltrating lymphocytes within the TME.

The goal of this small study was to demonstrate that CLARITY-processed specimens may enable a more accurate, unbiased analysis of tumor samples in comparison to conventional slide-based histology. As such, we first determined an observed 0.01% limit of detection in CLARITY processed cell pellet model using both manual and automated cell counting. We developed optimized antibody conditions to demonstrate specific staining of various cellular, membrane, and nuclear markers using CLARITY processed tissues including isotype controls, which have not previously been included in previous publications. We then applied the optimized conditions to clinical biopsy specimens, incorporated quantitative analysis of the 3D images, and compare them to their respective standard 2D images. We expounded our efforts through a comparison of clinical human breast cancer patients’ core needle biopsy tissues processed with the CLARITY technique and the standard FFPE block method by manual quantification of Ki67 post processing. The overall composite Ki67 manual score obtained from CLARITY-processed tissues was concordant with the respective FFPE slide results. This study demonstrated that CLARITY is not only compatible with various tissue processing preparations, allowing for prospective and retrospective analysis, but is also a powerful technique for the quantitative identification of select biomarkers that may identify tumor cell heterogeneity.

## Results

### A 0.01% limit of detection can be observed in CLARITY processed cell pellets

**Figure 1:**
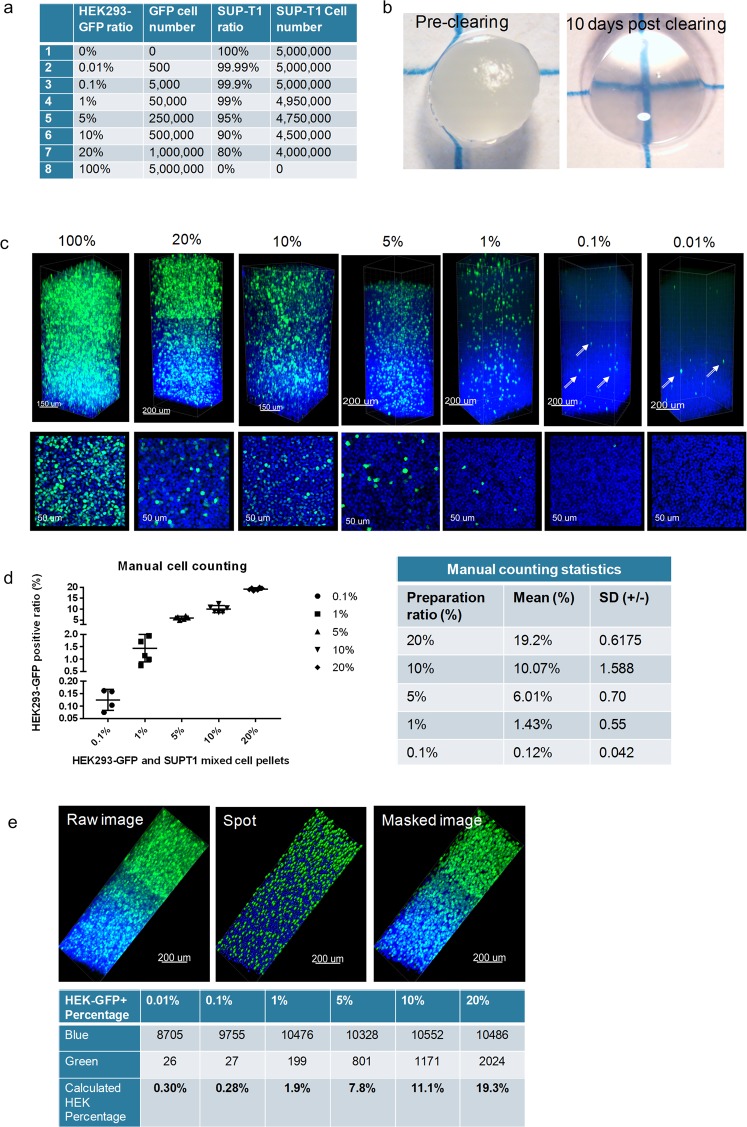
A 0.01% limit of detection can be observed in CLARITY processed cell pellets. (**a**) The mixed cell pellets ratios generated with HEK293-GFP and SUP-T1 cells. (**b**) A representative mixed cell pellet imaged before clearing and 10 days after clearing under a dissecting microscope. (**c**) Confocal microscopy (Leica SP8) images of the cell pellets at different ratios (25X). Top row: 3D z-stack images, Bottom row: a corresponding representative 2D optical section image. The HEK293-GFP cells (green) can be detected in the FOV for all ratio groups, including the rare event level of 0.01%. (**d**) Manual counting of HEK293-GFP (green) was accomplished by calculating the ratio of positive GFP cells/total DAPI (blue) cells (optical sections were used for manual counting, n ≥ 5 for each sample). Statistics were calculated by Microsoft Excel and GraphPad Prism 7.0. (**e**) The automated analysis and quantification of the GFP positive cell pellets was performed using a spot detection strategy generated with Imaris 9.1.2 software.

Given the heterogenous composition of the TME, the ability to detect rare cells within the cell population of a tissue has become a key element in assessing disease progression. A “rare event” in flow cytometry is usually defined by a detection frequency of 0.01% or lower within a select number of events^30^. While flow cytometry has several benefits, it is also a destructive technique that removes all spatial components of the TME. With these limitations in mind, we sought to determine if rare events could be detected with the CLARITY method using an *in vitro* approach. We created a mixed cell pellet model using HEK293-GFP (green fluorescent protein-endogenous expression) and SUP-T1 (unlabeled) cells at specific ratios (Fig. 1a). The endogenously expressed GFP signal minimized any bias and variables associated with immunostaining. All of the cell pellets were lipid-cleared in about 10–12 days (Fig. 1b). As shown in Fig. 1c, the fluorescent green signal of the HEK293-GFP cells was bright and easily detected in the cell pellet. This was confirmed with multiple fields of view (FOVs) for each group. Assessment of the cell pellet ratio accuracy was performed through a manual count of GFP positive cells using multiple individual optical sections, at least 50 µm apart in Z direction. With the exception of the 0.01% “rare event” group, all the ratios from 20% to 0.1% could be manually counted, and the GFP/SUPT1 cell ratio percentage matched the original cell preparation ratio (Fig. 1d). The 0.01% cell ratio demonstrated few positive GFP cells over the entire FOV thickness, and thus not all the optical sections had positive GFP cells to count. As a result, manual counting of the 0.01% group resulted in a higher-than-average false negative or false positive result. While it is possible to count the total number of “rare” GFP positive cells in the 3D FOV image, a manual method to quantify the total number of cells in a particular FOV is extremely difficult and taxing due to the inability to clearly track cells that extend into more than one optical section.

In fact, this specific concern with manual counting of the 0.01% group, underscored the necessity for an automated 3D image analysis and individual event segmentation that could not only eliminate duplicate cell counting, but also expedite the cell counting process. A preliminary experiment was employed on the same GFP cell pellet series to establish feasibility for automated cell counting. One FOV, defined as the entire imaged z-stack from each cell pellet, was utilized for the preliminary automated cell count. The spot feature in the Imaris software was activated, and parameters were established to distinguish the two different cell sizes of the HEK293 and SUP-T1 cell lines for the most accurate count of total cell nuclei. Since only the HEK293 cells expressed GFP, the parameters for GFP signal detection were based on a single cell size. A representative example of the imaging workflow is shown in Fig. 1e. The software generated a GFP positive cell count (green channel) and a total cell count based on the blue channel (DAPI), and the HEK-GFP + percentage was subsequently determined (Fig. 1e). Overall, we were able to establish feasibility for an automated counting workflow with a final count that was not significantly different from the manual count; however, at the lower extremes of the cell ratio the small variations appeared to be more impactful. A more comprehensive analysis employing multiple FOVs, similar to the manual counting approach, would be extremely beneficial to determine the consistency and standard deviation of our methodology.

### Establishment of an optimal antibody concentration for immunostaining CLARITY processed tissue

In traditional thin-section immunohistochemical (IHC) staining, antibodies are applied directly onto 5-µm thick tissues and typically incubated for 1–24 hours with minimal problems. However, when immunostaining thicker tissue sections (>100 µm), antibody penetration problems, resulting in a “sandwiched” staining pattern, are frequently observed^10^. There may be several reasons to explain this staining pattern, such as unoptimized antibody concentration or incubation time due to the slow passive diffusion of antibodies into the center of thick tissue sections. To this end, there was a need to establish a specific approach for immunostaining thicker tissues that would result in saturated and optimal antibody binding. To address this matter, we utilized two different types of tissues (MCF-7 xenograft tumors and hyperplasia human tonsils) and three different antibodies (pan-CK, Ki67, and CD3) for titration experiments. Pan-CK(AE1/AE3)-AF488 (a directly conjugated antibody) could bind homogenously to the epitopes in the MCF-7 tissues and serve as an ideal marker for an initial XZ scan by a confocal microscope. The samples’ XZ scans were quickly analyzed daily to obtain the depth of antibody penetration in relation to antibody concentration and incubation time (Supplemental Fig. S1). For the pan-CK antibody titration with MCF-7 xenografts, four different dilutions (1:5, 1:10, 1:20, 1:50) were tested and imaged daily for 10 days (Supplemental Fig. S1a). These initial data demonstrated a clear correlation between antibody concentration, penetration depth, and incubation time. The signal was found to reach maximum saturation on day 5, with no significant signal improvement observed with prolonged incubation or replenishment of the primary antibody (Supplemental Fig. S1b,c). An incubation period of five days was then applied to the remaining antibody titrations for Ki67 and CD3 in the human tonsil tissue, where the observed expression pattern and location was particularly specific.

**Figure 2:**
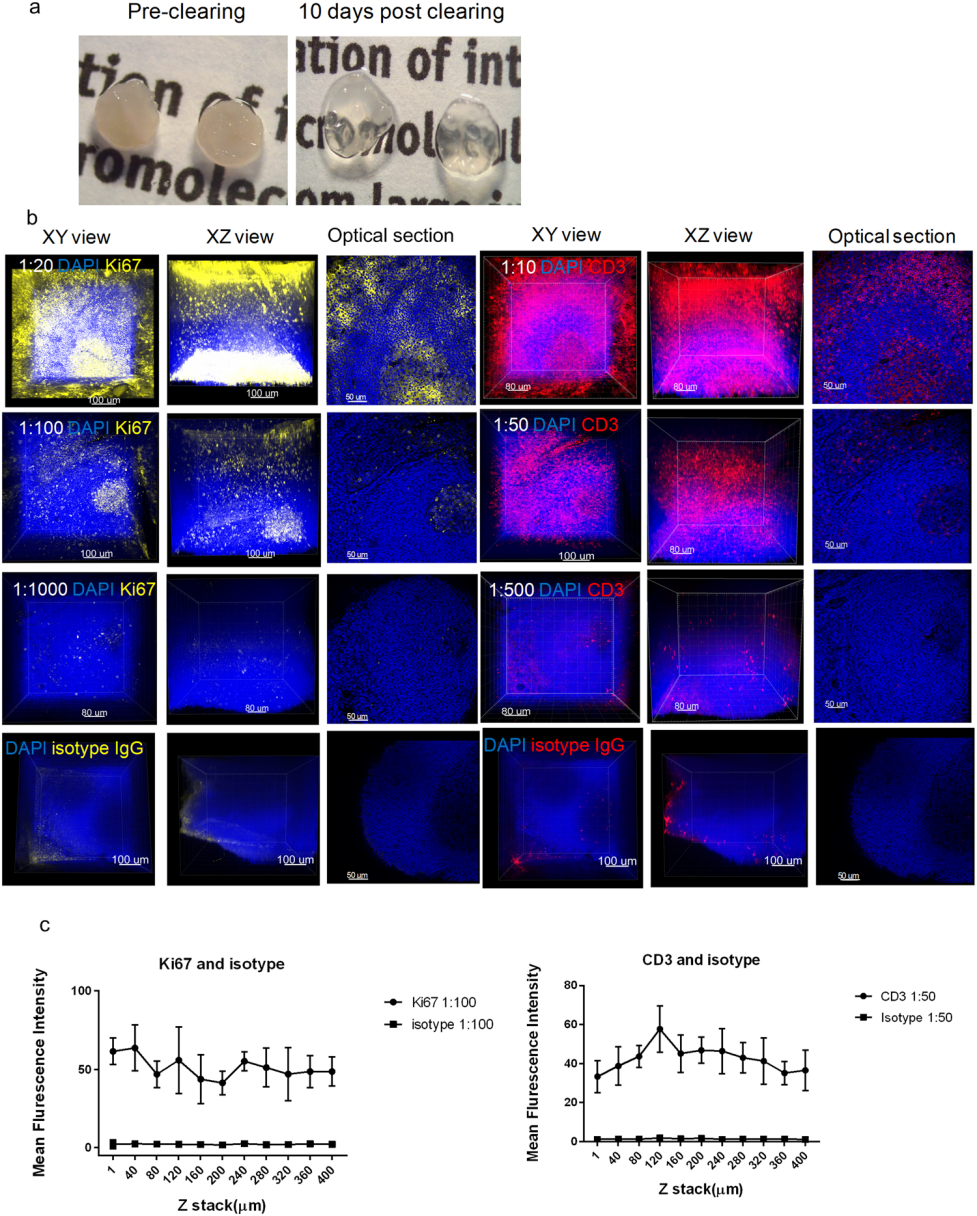
Establishment of an optimal antibody concentration for HM embedded tissues. (**a**) Images of human tonsil sections embedded in an A4B4P4 HM (3 mm × 3 mm × 0.5 mm) before and after clearing, 10 days. (**b**) Confocal z-stack imaging of antibody titrations in tonsil tissues (25X), Ki67 (yellow, left), CD3 (red, right), and DAPI (blue). For each antibody titration, three different concentrations were used: Ki67 (1:20, 1:100, 1:1000), CD3 (1:10, 1:50, 1:500), DAPI (1:500), and displayed in 3D: XY axes view (left), XZ axes view (middle), and a respective 2D optical slice (right). The images of corresponding isotype controls stained at the optimal concentrations are showed in the last row. (**c**) The mean fluorescence intensity (MFI) of Ki67 or CD3 and their isotypes were quantified using Image J at different tissue depths. Each optical section has multiple selected areas (n = 5) used for MFI quantification and the images were plotted with mean and standard deviation values by GraphPad Prism 7.0.

The human tonsil tissues, embedded in an A4B4P4 HM, were biopsy-punched to an equal size (3 mm × 3 mm × 0.5 mm) and cleared for approximately 10 days (Fig. 2a). The primary antibodies were titrated at the following dilutions: Ki67 (1:20, 1:100, 1:1000) and CD3 (1:10, 1:50, 1:500). Following immunostaining, each test group was then imaged using a z-stack scan (step size 2 µm) taken from the germinal center region for comparative analysis (Fig. 2b). We found that a concentration of 1:100 and 1:50 for the Ki67 and CD3 antibodies, respectively, demonstrated the optimal titration with a low background, high signal-to-noise (S/N) staining, and complete antibody penetration. At the lowest concentration, Ki67 (1:1000) and CD3 (1:500), both had a weak signal throughout the entire tissue, indicating an insufficient antibody concentration for the amount of epitope present in the tissue. On the other hand, tissues immunostained at the highest concentrations, Ki67 (1:20) and CD3 (1:10), showed a low S/N, demonstrated by high background and non-specific staining, particularly on the tissue surface. We further compared the mean fluorescence intensity (MFI) of the antibody concentrations, Ki67 (1:100) and CD3 (1:50) to their respective isotype controls using ImageJ. Optical sections, spaced 40 µm apart in the Z direction were selected for analysis, and both antibodies demonstrated consistent MFI signals throughout the thickness of the tissue (Fig. 2c).

### The incorporation of the CLARITY process on previously fixed clinical core needle biopsy samples preserves the tumor microenvironment

**Figure 3:**
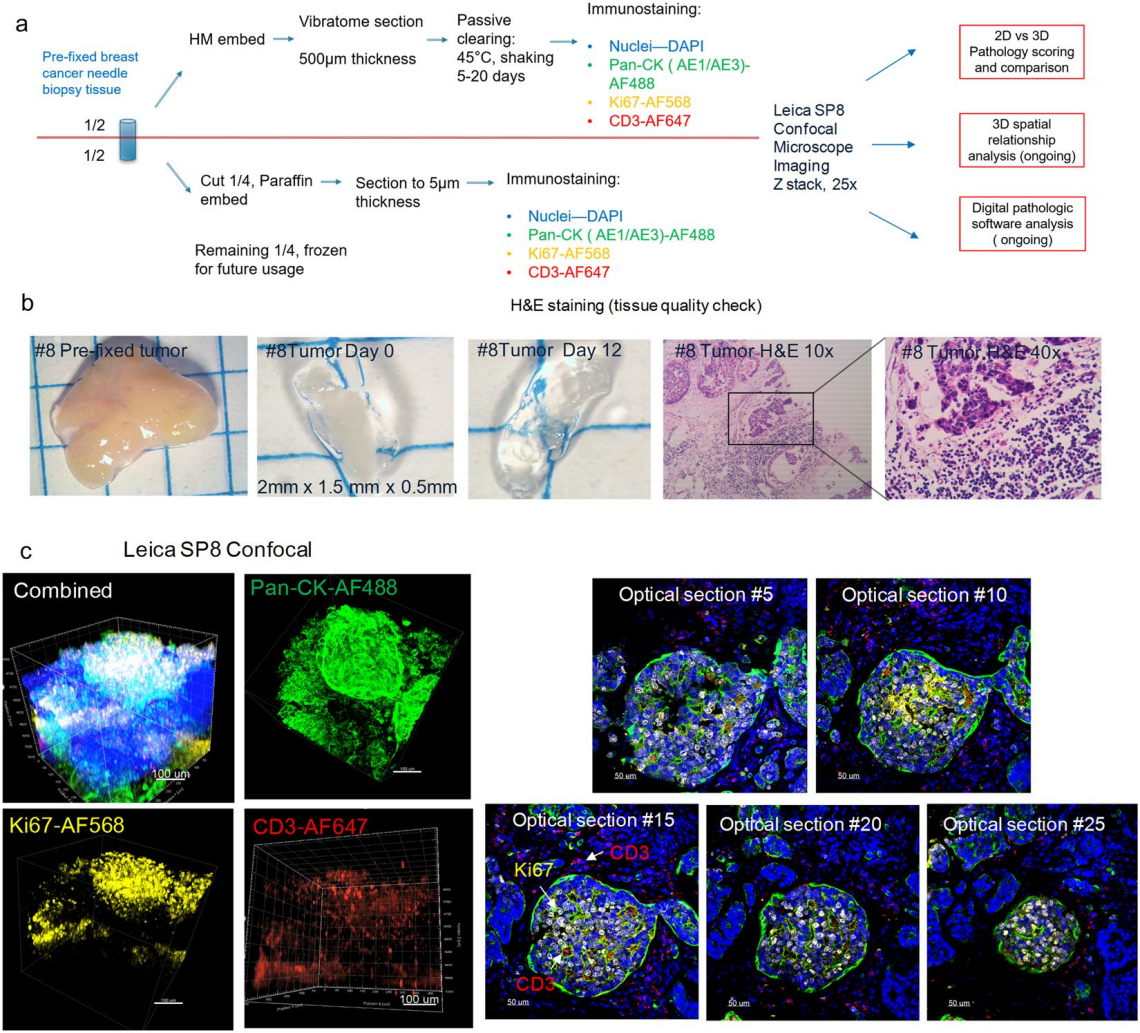
The incorporation of the CLARITY process on previously fixed breast cancer core needle biopsy samples preserves the tumor microenvironment. (**a**) A schematic workflow diagram of tissue processing, clearing, immunostaining, and imaging for the pre-fixed clinical biopsy samples. (**b**) Gross image of tumor biopsy tissue #8 before vibratome sectioning (2 mm × 1.5 mm), after sectioning (0.5 mm thickness), and post lipid clearing. Representative images from the FFPE corresponding tissue were also H&E stained and displayed (10x, 40x). (**c**) Confocal microscope imaging of breast cancer tumor #8 (25X). The total 3D volume is visualized in individual and merged channels (left group), and 2D optical section images (25X, right group) demonstrate the staining uniformity that is maintained and increasing imaging depths. The breast tissue was immunostained with antibodies against pan-CK (green), Ki67 (yellow), CD3 (red), and a DAPI nuclear counterstain (blue). Images were visualized by Imaris Version 9.1.2.

We expanded our studies into previously formalin-fixed human breast cancer core needle biopsy tissue from patients that underwent excisional surgery. As previously mentioned, the samples were collected, sectioned into mirrored pieces for subsequent A4B4P4 HM-embedding (500 µm sectioned tissue), and FFPE block tissue preparation (5 µm sectioned tissue) followed by immunostaining and analysis (Fig. 3a). The samples not only remained intact, but cellular morphology was preserved suggesting that a pre-fixation step following by hydrogel embedding and immunostaining could be a feasible workflow.

The average lipid-clearing time for the breast cancer tissues ranged from 5–14 days depending on the varying tissue compositions of individual samples. The majority of the samples reached optical transparency; however, some specimens contained fibrotic regions and retained some opacity (Fig. 3b). The samples demonstrated specific staining of various cellular and nuclear markers. Antibody signals, from all four laser channels, were observed throughout the whole tissue thickness using laser-gain adjustment through the z-stack, with no observed “sandwich” staining pattern (Fig. 3c, Supplemental Videos S1–S3), confirming full sample penetration. A sample of individual optical sections, from tumor #8, were selected to further demonstrate the specific subcellular staining observed throughout these thick tissue sections.

### CLARITY allows for an unbiased pathology score based on Ki67 expression

**Figure 4:**
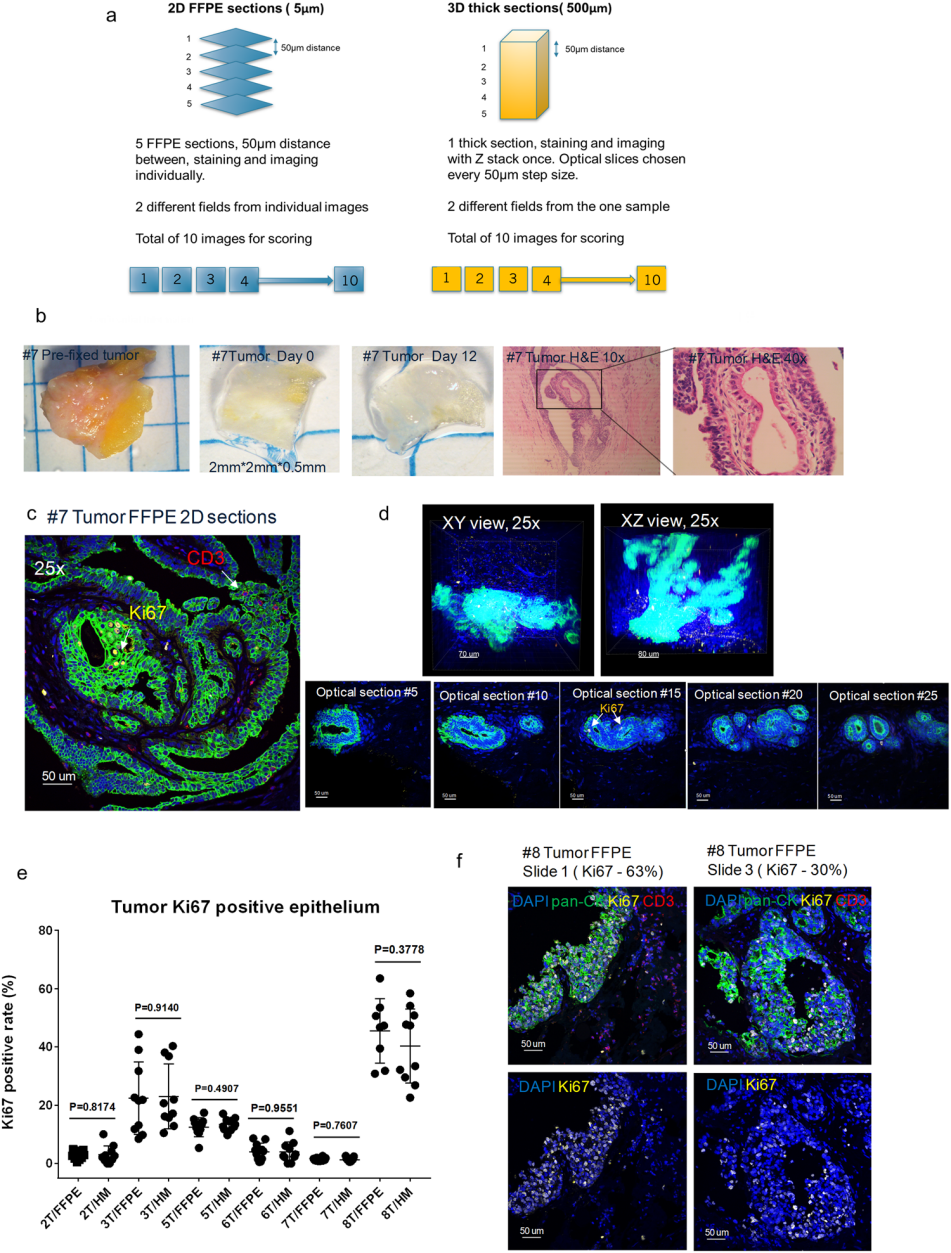
CLARITY allows for an unbiased pathology score based on Ki67 expression. (**a**) Schematic diagram depicting the methodology and optical slice selection used to compare individual 2D FFPE sections (5 µm, left) to the corresponding 3D thick section (500 µm, right) for Ki67 scoring. Blind scoring was done by two independent pathology professionals and results were analyzed with GraphPad Prism 7.0. (**b**) Images of tumor tissue #7 before sectioning, after sectioning, post clearing, and the corresponding FFPE H&E staining of the tissue (10x, 40x). Representative confocal images of tumor #7 (**c**) 2D FFPE immunofluorescence staining (25X) and (**d**) 3D volumetric (top row) and optical slices (bottom row) HM embedded thick tissue immunofluorescence staining (25X). Blue: DAPI, Green: pan-CK, Yellow: Ki67, Red: CD3, 25X (**e**) A comparison of Ki67 positive ratio (Ki67 positive epithelial/total epithelial) in corresponding FFPE thin sections and HM thick sections (p-values as listed, unpaired t test) in biopsy samples. (**f**) Representative images of tumor #8 with heterogenous expression of Ki67 in FFPE 2D thin sections from different sections (25X) demonstrating differing levels of Ki67 positive rates.

To substantiate that the antibody signal detected was representative of its expression level, we utilized a traditional method to score Ki67 expression in our thick HM-embedded tissue images in comparison to the gold standard FFPE. Using the previously mentioned FFPE thin sectioned tissues, at least 10 images from five individual FFPE slides (50 µm apart) were chosen to score. Additionally, optical sections from the z-stacks of the thick tissue sections were selected in a similar fashion to score (Fig. 4a). An example of breast tumor #7 was used to demonstrate the approach. Based on the H&E images, tumor #7 was determined to be an invasive ductal carcinoma with some regions appearing to be adjacent normal tissue within the same block (Fig. 4b). Due to its heavy fibrotic nature, only partial tissue clearing was achieved (Fig. 4b). The stained FFPE slides and thick tissue sections were both imaged with a confocal microscope (25x objective) and showed specific staining of Ki67, pan-CK, and CD3 with the high resolution required for pathology scoring (Fig. 4c,d, Supplemental Video S2). The comparison of the overall Ki67 pathology scoring data between the 3D thick tissue images and 2D FFPE images from multiple samples revealed there was no statistical difference between the two tissue processing methods (Fig. 4e). However, it is worth mentioning that certain tumor tissues, such as tumors #3 and #8, were found to be more heterogenous than the others (Fig. 4f). Despite a comparable cumulative mean Ki67 score between multiple thin FFPE slides and optical sections from the thick HM-embedded tissue (~30–60%), we found using one randomly chosen slide from the FFPE slide group would not be a true representative of the overall molecular expression level. However, the advantage of employing the non-destructive CLARITY method with thick tumor tissues, is the ability to evaluate an entire tumor and obtain an unbiased Ki67 pathology score that truly reflects the overall molecular expression levels.

### CLARITY processed tissues can be incorporated for retrospective analysis of FFPE tissues

**Figure 5:**
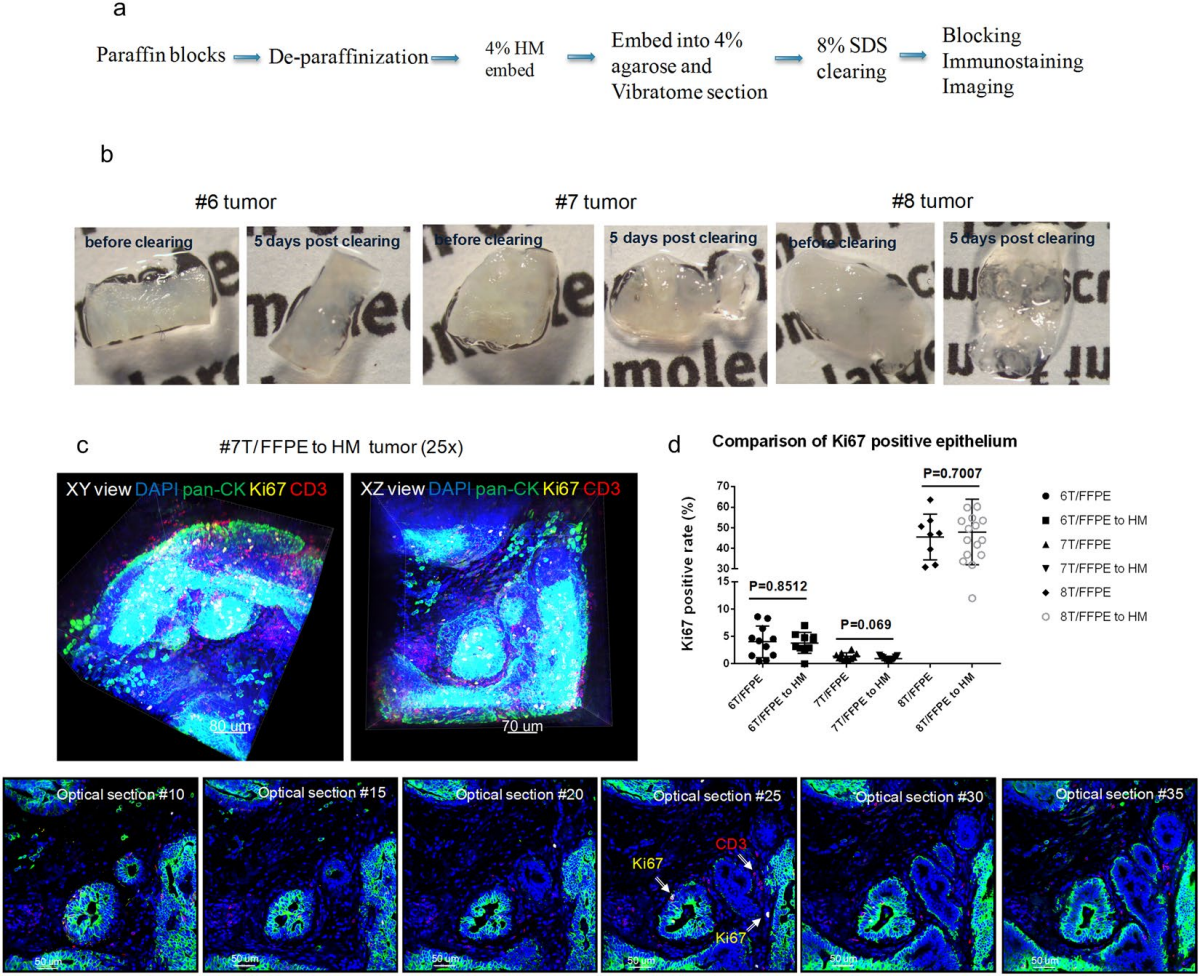
CLARITY processed tissues can be incorporated for retrospective analysis of FFPE tissues. (**a**) A general workflow diagram of deparaffinization and subsequent tissue clearing and immunostaining utilized for archived FFPE blocks (**b**) Deparaffinized FFPE tumors #6, #7, and #8 (Indiana breast cancer core biopsy tissue) were re-embedded into an A4B4P0 HM solution followed by lipid clearing. The images show the tissues both before and five days after lipid clearing. (**c**) Confocal images of tumor #7 at 25X (Blue: DAPI, Green: pan-CK, Yellow: Ki67, Red: CD3). Top row: 3D volumetric images viewed from both the XY axes and XZ axes. Bottom row: Individual 2D optical section from the 3D view from increasing depths. In certain optical sections, the Ki67 positive epithelial cells and infiltrating CD3 positive T cells can be observed, denoted by arrows. (**d**) A comparison of Ki67 positive ratio (Ki67 positive epithelial cells/total epithelial cells) scores between respective converted FFPE to HM and FFPE thin sections (unpaired t test, p-value as listed).

CLARITY has already demonstrated compatibility with multiple pre-clinical and clinical tissue types preserved in a variety of ways (fresh, frozen, and formalin-fixed); however, the applicability of CLARITY to archived specimens has not been well studied. We investigated whether archived tissues from FFPE blocks could be processed by the CLARITY method, immunostained, and manually quantified. Feasibility was first demonstrated using normal mouse kidneys and MCF-7 xenograft tumors taken from FFPE blocks. The paraffin blocks were melted, the tissues were deparaffinized, and embedded with an A4B4P4 HM solution (Fig. 5a). The kidneys and xenograft tumors were then cut into 200 µm thick slices that were cleared, immunostained, and imaged to detect multiple biomarkers (α-SMA, lectin, pan-CK and Ki67) (Supplemental Fig. S2).

Additional confirmation of our findings was done by utilizing clinical FFPE samples. We used a portion of the previously described FFPE breast cancer core needle biopsy tissues (specifically tumors #6, #7, #8) to deparaffinize, HM-embed, and immunostain. These FFPE-to-HM converted samples were then compared to their respective standard FFPE 2D thin sections after they were scored for Ki67 expression. All of these tissues maintained their structure during the workflow process, and the lipid-clearing duration was not affected by the previous FFPE sample preservation (Fig. 5b). We were able to confirm our initial findings, reflected through robust immunostaining with antibodies against pan-CK, Ki67, and CD3 with a DAPI counterstain (Fig. 5c, Supplemental Video S4). Finally, we performed an analysis on the Ki67 score generated for both the FFPE-to-HM tumors and their mirror-faced FFPE 2D thin section respective counterparts (Fig. 5d). In general, the Ki67 expression for both preparations were found to be equivalent, which indicated CLARITY can be compatible with archived FFPE tissues. Tumors 6 and 7 both had minimal intra-tumor variation in Ki67 expression (approximately 0–10%); however, the most impactful result from these samples was highlighted in tumor #8, which was found to have a wide-range in expression of Ki67 positive cells (~30–60%).

## Discussion

The practical limitations of conventional histology include labor-intensive and imprecise sample sectioning, reconstruction, and quantification of biomarkers^31,32^. Furthermore, the majority of clinical IHC testing involves single analyte detection visualized in a small number of representative samples, hindering the ability to understand inherent spatial heterogeneity in tumors^33^. In addition, there can be variability in an FDA approved or laboratory derived tests depending on the antibody/assay used, which can lead to further discrepancies^34,35^. To address these needs, newer methods that employ multiplex immunofluorescence have been applied to 2D FFPE tissue to partially overcome the single analyte approach, and to add spatial and phenotypic information^36,37^. While advancing research applications in the field of immuno-oncology, these methods are still based on a small sampling of the tumor. CLARITY could become a technique that can address both the spatial heterogeneity of intact tumors and multiplex staining directly, holding the unique advantage in immune microenvironment research. Furthermore, optimization of key parameters could lead to future quantitative spatial relationships that are clinically meaningful that may overcome the limitations of 2D thin section techniques.

Unlike the lipid-rich and homogeneous structure of the mouse brain, solid tumors often have an abundance of fibrotic tissue and a heterogeneous composition, which can make a visual assessment of tissue clearing difficult between individual samples. However, there remains a need for multiplexed analysis of key biomarkers in cancer pathological specimens, whereby complex spatial patterns of cells, as well as tissue architecture can be reproducibly measured. A standardized, reproducible tissue processing method that allows the accurate identification, as well as quantification of key biomarkers and spatial relationships within the TME in 3D for research and clinical applications is paramount. As a result of all these unique factors and requirements, a comprehensive optimization of key aspects of the CLARITY technique is necessary to achieve these goals.

While previous publications have addressed multiple ways to improve antibody penetration and staining quality through either optimization of the HM formulation, tissue fixation^9,10,24^, or by applying active antibody infusion methods^22,24,38^, we sought to understand whether full penetration of select molecular probes could be achieved using an antibody titration approach. In the titration study, we decided to utilize a tissue model given the divergent antibody penetration dynamics between cell pellets, which lacks an extracellular matrix component, and structurally intact tissues. Murine xenograft tumor tissue, although moderately different from human tumor tissues, can serve as a robust model to study antibody titration/penetration for tumor markers. Contrary to typical 2D antibody titration, the antibody penetration necessary for thicker HM-embedded tissues requires analysis in the axial direction as well. Thus, an ‘XZ’ microscopic scan was required to assess penetration in the Z axis, followed by a calculation of the S/N ratio. The primary objective was to establish the appropriate antibody concentration range that exhibited both a high S/N ratio and maximum penetration and saturation of the thick HM-embedded sections. The more complex and heterogenous human tonsil was deemed more appropriate to titrate the remaining antigens, Ki67 and CD3, which required a detailed subcellular and cellular analysis, respectively, on individual ‘XY’ optical sections prior to Z axis analysis. We opted to focus on the originally published CLARITY HM formulation^8^ to substantiate the necessity of proper antibody titration for thick tissue immunostaining penetration. Furthermore, we also included isotype control groups, with matched characteristics (species, class, type) to their respective primary antibodies, to provide a more accurate negative control in thick tissue immunostaining which has been neglected in previous publications.

As part of this study, we set out to better understand if the CLARITY technique could aid in the detection of rare events in a 3D volume. While we were able to correlate the data between the theoretical and actual groups, especially in “frequent events” (0.1–20% GFP samples), we encountered some difficulties in providing similar evidence for the 0.01% “rare event” group. Manual counting can be a laborious and time-consuming approach, particularly in the case of rare event counting over a 3D volume of tissue^39^. In contrast, digital image analysis can be a quick, non-labor-intensive approach; however, due to the general parameters established for counting, it can result in increased false positives until the system is optimized for the particular data set of interest. Although there were some inherent variables in cell preparation and cell counting, we did demonstrate an ability to detect true positive rare events using an endogenous fluorescence marker within a volume of tissue. Being able to detect rare events, while maintaining the spatial structure of the tissue has implications for understanding the complex biology of a heterogeneous system more accurately then 2D thin section FFPE methods.

Further examination of key protein measurements using CLARITY methods was determined using multiplex immunostaining and a manually derived Ki67 count in human breast cancer specimens. For comparison to gold standard methods, we utilized a composite of 2D FFPE thin section specimens from another portion of the patient’s core biopsy. In our studies, we demonstrated the potential for differential expression of Ki67 expression between thick CLARITY processed and serial thin FFPE prepared tissue sections. The concordant results indicated that the clinical samples processed by the CLARITY method, had similar quantitative results compared to the gold standard methodology when compared as a composite. Importantly, significant epitope loss was not observed when compared with standard 2D thin section results. Additional validation studies with a large dataset will be required to confirm our findings. Interestingly, the results from a small set of archived FFPE tissue samples from the same patients that were subsequently processed by the CLARITY method were also concordant with tissues that were prepared by traditional FFPE techniques. This suggests that in addition to the CLARITY method being compatible with fresh, frozen, and fixed tissue, it is also compatible with archived FFPE tissue. This opens up the ability to process archived FFPE samples and compare the results with clinical outcome data.

Understanding the intricacies of both inter-tumor and intra-tumor heterogeneity have become an important concern in establishing prognostic and predictive molecular biomarkers in clinical oncology^4–7^. There are currently very few clinical biomarkers that correlate with clinical outcome and confounding factors such as intra-tumor heterogeneity can impact the accuracy of these biomarkers^4,6^. The need for more effective prognostic and predictive biomarkers for immuno-oncology drugs is growing rapidly, as response rates continue in the 10–30% range and the extent of possible drugs, including combination therapy, grows at an accelerating pace^40^. Sampling bias can result from sparse observation of a single FFPE slide, which can impact the reliability of the scoring analysis. Therefore, 3D techniques, such as CLARITY, clearly hold the advantages of enabling a more accurate and quantitative analysis of tumor samples, particularly for heterogeneous samples.

Although non-destructive tissue techniques, such as CLARITY, have the ability to preserve structural integrity of the tissue and reveal biomarker relationships in 3D, limitations still exist. The major limitation is the length of time it takes to clear and immunostain tissues. Lengthy tissue processing time frames limit the turnaround time of data to be a viable technology for use in drug development and clinical research applications in the future. Limitations also exist with the commercially available image analysis software, and to that end, more sophisticated and applicable software must be developed to handle large datasets, as well as the development of machine learning algorithms to handle feature extraction and meaningful 3D spatial relationships in the TME. To this end, automated and expedited ways to process tissue in 3D as well as automated imaging is critical for this technique to be useful for both clinical research applications as well as integration into typical clinical workflow paradigms.

In conclusion, our study demonstrated that CLARITY prepared tissues are compatible with various processed clinical tissues, thus allowing future prospective and retrospective analyses. It is a powerful technique to quantitatively identify biomarkers in the TME using a manual scoring approach. This manuscript would be the first example in CLARITY processed tissue for antibody titrations and optimization, in conjunction with a comparative isotype control. Based on these studies, the ability to accurately quantify the markers in CLARITY processed tissue holds great promise that the TME can be more accurately evaluated with larger volumes of intact tissue. The development of automated image analysis methods that can be incorporated into the 3D analysis of preclinical and clinical tissue is crucial as well.

## Methods

### Animals and human specimens

Normal murine tissues and xenograft model MCF-7 tumors were generated and collected in the Molecular Medicine Research Institute (MMRI, Sunnyvale, CA) animal facility based on guidelines of the National Institutes of Health (NIH) and approved by the Institutional Animal Care and Use Committee (IACUC, protocol #16-002). Approximately 10^7^ MCF-7 (ATCC HTB-22, Manassas, VA) cells were utilized to inoculate NOD *scid* gamma female mice (6–8 weeks, #005557, The Jackson Laboratory, Bar Harbor, ME) to generate tumors.

Human de-identified breast cancer core needle tissues from excision biopsies (n = 6) were collected from the Indiana University School of Medicine. All protocols were reviewed and approved by the Institutional Review Board (IRB) of Indiana University. Samples and clinical records were anonymized prior to access by the authors and linked with a numerical identifier. The requirement for informed consent was waived by the IRB. Tissues were pre-fixed in 10% neutral buffered formalin for less than 24 hours and shipped in RPMI-1640 media. Upon arrival, individual tissues were sectioned as follows: one-half was embedded into a HM for thick section (500 µm) processing, one-quarter (a “mirror face” to the HM-thick section) piece was paraffin-embedded using a Tissue Tek VIP 1000 processor for subsequent thin section (5 µm) analysis, and the remaining one-quarter of the tissue was stored at −80 °C for future use. Fresh frozen human de-identified hyperplastic tonsil tissue was obtained commercially from Asterand (Detroit, MI).

### Cell pellet preparation and processing

Cell pellet mixtures were generated by using human embryotic kidney cells 293-green fluorescence protein (HEK293-GFP) stable cells (#SC001, GenTarget, Inc., San Diego, CA) and SUP-T1 (#CRL-1942, ATCC, Manassas, VA). The cell pellets were embedded into the classic CLARITY A4B4P4 HM formulation for 48 hours at 4 °C. Each cell pellet contained approximately 5 × 10^6^ cells, with a cell pellet size measuring 3 mm × 3 mm × 2 mm. The A4B4P4 hydrogel, which has been previously described was composed of 4% acrylamide (vol/vol), 0.05% bis-acrylamide (vol/vol), 4% PFA (vol/vol), and 0.25% VA-044 initiator (wt/vol) in phosphate-buffered saline (PBS)^9^. The cell pellets were degassed with a nitrogen-flush and vacuum chamber for 10 minutes at room temperature (RT) followed by polymerization at 37 °C for three hours. After removing the excess gel, the cell pellets were submerged into a clearing solution containing 8% sodium dodecyl sulfate (SDS, vol/vol) and sodium borate buffer (200 mM, pH 8.5), and placed in a shaking incubator at 45 °C for lipid clearing until they reached optical transparency. The pellets were removed from the SDS/borate clearing solution, rinsed 3–4 times with PBST (0.1% Triton X-100 (vol/vol) in PBS) over a 24-hour period, and stained with DAPI (#62248 Thermo Fisher Scientific, 1 mg/ml, 1:200, Waltham, MA) for three days at 37 °C. Finally, the pellets were washed with PBST 4–5 times overnight, before submersion into RapiClear CS (#RCC, S002, Sunjin Labs, Taiwan), a refractive index (RI) matching solution. RI matched cell pellets were mounted onto a glass coverslip (22 × 30 mm, #72200-20 Electron Microscopy Sciences, Hatfield, VA) and imaged in a z-stack with a Leica SP8 confocal microscope system (Leica Microsystems, Buffalo Grove, IL) using a 25X, 0.95NA water immersion objective (5 µm z-step size, 1024 × 1024 resolution). The cell pellets were excited by both the 405 nm and 488 nm laser lines and multiple fields of view (FOVs) were imaged. The images were reconstructed in 3D and analyzed using Imaris 9.1.2 software (Bitplane, Zurich, Switzerland). For manual cell counting, the GFP positive cells and total cells, based on the DAPI staining, were counted from at least five different optical sections (at least 50 µm apart, along the z-axis, to avoid duplicate single cell counting) and data were input into Excel and GraphPad Prism 7.0 for statistical analysis.

### Tonsil tissue processing and antibody titration

The tonsil tissue was embedded into an A4B4P4 hydrogel and cleared as described previously for the cell pellets. Ki67 (1:20, 1:100, 1:1000) and CD3 (1:10, 1:50, 1:500) antibodies were applied at 37 °C for five days followed by the Alexa-Fluor secondary antibodies incubated under the same conditions. After washing with PBST and RI matching, images were taken with the Leica SP8 confocal microscope using a 25X, 0.95NA water immersion objective (3 µm z-step size, 2048 × 2048 resolution, with laser adjustment (1% to 20%) to account for light scattering at increased imaging depth). Tissues immunostained with either specific biomarkers or with isotype controls were imaged with exactly the same settings.

### Pre-fixed human breast cancer core needle biopsy tissue processing

Upon arrival, the prefixed breast cancer core needle biopsy tissues (2 mm × 2 mm × 1 cm) were rinsed with PBS once, sectioned into “mirror-faced” halves with one-half placed into the A4B4P4 HM formulation for 48 hours at 4 °C. Then the tissues were degassed and polymerized as previously described for the cell pellets. Afterward, the tissues were embedded in a 4% agarose mold, sectioned into 200 µm or 500 µm thick sections with the Leica VT1200S vibratome (Leica Biosystems, Wetzlar, Germany), and cleared as previously described for the cell pellets. Briefly, the samples were blocked (10% normal goat serum, 3% bovine serum albumin, 0.1% Triton X-100, 0.01% sodium azide (wt/vol), PBS) overnight at RT, incubated with primary antibodies diluted in PBST at 37 °C for five days, washed five times with PBST for one hour each, and incubated with Alexa Fluor-conjugated secondary antibodies diluted in PBST at 37 °C for five days. After a PBST wash, the samples were RI matched, and imaged. The antibodies and dilutions used in this study can be found in Table 1. The samples were RI matched and imaged with a Leica SP8 confocal microscope using a 25X, 0.95NA water immersion objective (3 µm z-step size, 2048 × 2048 resolution).

**Table 1:** Antibodies and dilutions employed in the noted studies.

Ab name	Clone	Dilutions	Vendor	Catalogue #	Stock concentration
**Pre-fixed and FFPE processed human breast cancer biopsy tissue staining (500 µm)**					
Pan-CK-AF488	AE1/AE3	1:10	Thermo Fisher Scientific, Waltham, MA	#53-9003-82	0.5 mg/ml
Ki67	polyclonal	1:100	Abcam, Cambridge, UK	#ab15580	1.0 mg/ml
CD3	LN10	1:50	Leica, Wetzlar, Germany	#ACI3152C	32 µg/ml
Mouse IgG1-AF488 isotype control	MOPC-21	1:4	Biolegend, San Diego, CA	#400129	0.2 mg/ml
Rabbit IgG isotype control	polyclonal	1:20	Abcam, Cambridge, UK	#ab27478	0.2 mg/ml
Goat-anti-mouse AF647	polyclonal	1:200	Abcam, Cambridge, UK	#ab150115	1.96 mg/ml
Goat-anti-rabbit AF568	polyclonal	1:200	Abcam, Cambridge, UK	#ab175695	2 mg/ml
**FFPE processed mouse kidney and xenograft tissue staining (500 µm)**					
Αlpha-smooth muscle actin (α-SMA)-FITC	polyclonal	1:100	Abcam, Cambridge, UK	#ab188498	1 mg/ml
Lectin-Texas Red	N/A	1:1000	Vector labs, Burlingame, CA	#TL-1176	1 mg/ml
**Human breast cancer FFPE thin section staining (5 µm)**					
Pan-CK-AF488	AE1/AE3	1:500	Thermo Fisher Scientific, Waltham, MA	#53-9003-82	0.5 mg/ml
Ki67	polyclonal	1:400	Abcam, Cambridge, UK	#ab15580	1.0 mg/ml
CD3	LN10	1:500	Leica, Wetzlar, Germany	#ACI3152C	32 µg/ml
Mouse IgG1-AF488 isotype control	MOPC-21	1:200	Biolegend, San Diego, CA	#400129	0.2 mg/ml
Rabbit IgG isotype control	polyclonal	1:80	Abcam, Cambridge, UK	#ab27478	0.2 mg/ml
Goat-anti-mouse AF647	polyclonal	1:500	Abcam, Cambridge, UK	#ab150115	1.96 mg/ml
Goat-anti-rabbit AF568	polyclonal	1:500	Abcam, Cambridge, UK	#ab175695	2 mg/ml

### CLARITY-processed FFPE tissue preparation

FFPE tissues (normal mouse kidneys, MCF-7 xenograft tumors, and human breast cancer biopsies) were deparaffinized in xylene (3 exchanges: 2x, 1 hour each, and 1X- overnight) at RT, rehydrated in serial ethanol (EtOH) solutions (100% EtOH: 2x, 30 mins each; 95% EtOH: 30 mins; 70% EtOH: 30 mins; 50% EtOH: 30 mins), rinsed with cold tap water, and stored in a hydrated condition. The tissues were embedded into an A4B4P4 HM (mouse kidneys and MCF-7 xenograft tumors) or an A4B4P0 (pre-fixed human breast tumor, 4% acrylamide and 0.05% bis-acrylamide) at 4 °C for 48 hours and vibratome sectioned into 200 µm and 500 µm thick sections using a 4% agarose mold. The sectioned tissues were processed for lipid-clearing as described previously. Antibody dilutions are listed in Table 1.

### FFPE thin section tissue processing, immunostaining and H&E staining

FFPE thin sections (5 µm) were deparaffinized in xylene (2X, 10 min each) and rehydrated in an EtOH/water gradient series (100% EtOH - 10 min, 95% EtOH - 5 min, 70% EtOH - 5 min, 50% EtOH - 5 min). The rehydrated slides were briefly washed with water and kept hydrated. Antigen retrieval was performed using Diva Decloaker (#DV2004MX, Biocare Medical, Pacheco, CA), which included boiling the samples for 15 mins followed by cooling to RT (~10 minutes). The slides were then washed three times in PBS (10 mins each) and treated with Sudan Black B (#199664, Sigma-Aldrich, St. Louis, MO) for 1.5 hours, to quench autofluorescence. After washing with PBS three times (10 mins each), the slides were blocked with serum free protein block (#X0909, Dako/Agilent, Carpinteria, CA) for one hour at RT. Primary antibodies were incubated overnight at 4 °C, washed three times (10 mins each), followed by a secondary antibody incubation for one hour at RT. See Table 1 for the specific antibody dilution used. DAPI was applied as a nuclear counterstain at a dilution of 1:500 (500 µg/ml). The slides were mounted with Fluoroshield histology mounting medium (F6182, Sigma Aldrich, St. Louis, MO), covered with a glass coverslip (#48404-452, VWR, Radnor, PA), sealed with clear nail polish, and stored in the dark at 4 °C until imaged. For each breast cancer tissue FFPE block, at least one thin section (5 µm) was stained with hematoxylin and eosin (H&E) (Hematoxylin, #HHS128-4L; Eosin Y, #HT110316, Sigma, St. Louis, MO) and observed under a light microscope to verify the quality of the tissue.

### Pathology score and comparison on 2D FFPE thin section images and 3D CLARITY processed thick section images

Ki67 positive epithelial ratios were obtained for both 2D FFPE thin section images and optical slides from 3D CLARITY processed thick tissue. For the CLARITY processed thick sections, five optical sections from the 3D z-stack (50 µm apart, two FOVs, 10 total images) were taken for quantification. Ratios from the 2D FFPE thin sections used five slides (~50 µm apart), from each individual tumor for analysis. For example, slides #11, #21, #31, #41, #51 from tumor #7 and slide #2 (isotype control) were chosen to score. Two different FOVs from these six slides were chosen to score (total of 12 images). The ratio of Ki67 positive signal was calculated by the number of Ki67 positive epithelial cells/the total number of epithelial cells (at least 500 cells were counted per view, Fig. 4a)^29,41^.

### Statistics

The group mean, standard deviation (SD), and statistical significance were calculated using Microsoft Excel and GraphPad Prism 7.0. The p-value was calculated based on the unpaired t-test.

### Ethics approval and consent to participate

Human de-identified breast cancer core needle tissues from excision biopsies were collected from the Indiana University School of Medicine in accordance with guidelines from NIH and approved by the Institutional Review Board (IRB)

Normal murine tissues and xenograft model MCF-7 tumors were generated and collected in the Molecular Medicine Research Institute (MMRI, Sunnyvale, CA) animal facility based on guidelines of the National Institutes of Health (NIH) and approved by the Institutional Animal Care and Use Committee (IACUC).

## Supplementary information

Supplemental Information

Video S1

Video S2

Video S3

Video S4

## Data Availability

The datasets used and/or analyzed within the current study are available from the corresponding author upon reasonable request.
