# Supplementary material for: Three-dimensional imaging and quantitative analysis in CLARITY processed breast cancer tissues
Source: Sci Rep. 2019 Apr 4;9:5624. doi: 10.1038/s41598-019-41957-w (PMC6449377; doi:10.1038/s41598-019-41957-w)
Supplement: Supplementary file 1 — Supplemental Information [file 41598_2019_41957_MOESM1_ESM.pdf]

Supplemental Information

Three-dimensional imaging and quantitative analysis in CLARITY processed breast cancer tissues

Yi Chen[1], Qi Shen[1], Sharla L. White[1], Yesim Gokmen-Polar[2], Sunil Badve[2], *Laurie J. Goodman[1]

Authors Affiliations:

[1] ClearLight Biotechnologies, LLC (formerly known as ClearLight Diagnostics, LLC)
428 Oakmead Pkwy, Sunnyvale, CA 94085, USA
[2] Department of Pathology and Laboratory medicine, Indiana University School of Medicine, Indianapolis, IN 46202, USA

* To whom correspondence should be addressed:

Laurie J. Goodman, PhD
ClearLight Biotechnologies, LLC
428 Oakmead Pkwy, Sunnyvale, CA 94085
Phone: 650-245-8410
Email: lgoodman@clearlightbiotech.com

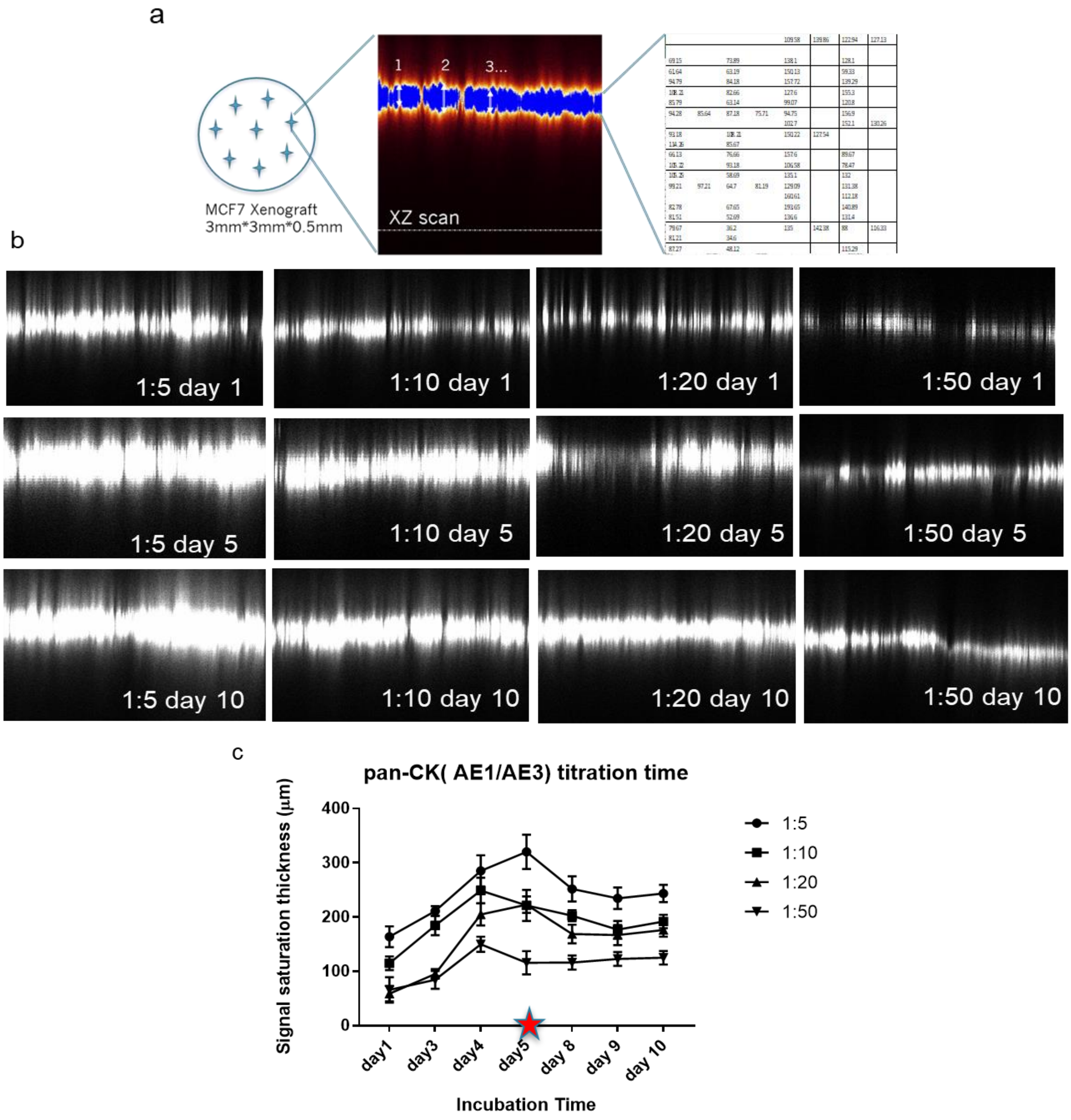

Figure S1: Pan-CK(AE1/AE3) titration in HM embedded MCF7 xenograft tissue (500 μm sections). (a) Schematic diagram of multiple FOVs method used to image and quantify pan-CK-AF488 signals utilizing the XZ scan in MCF7 tumor tissues. (b) Representative XZ scan images of pan-CK signal at different concentrations (1:5, 1:10, 1:20, 1:50) and time points (day 1, day 5, and day 10) by confocal microscope. (c) A plot of signal saturation over time (mean ± SD) with varying concentrations of pan-CK antibody.

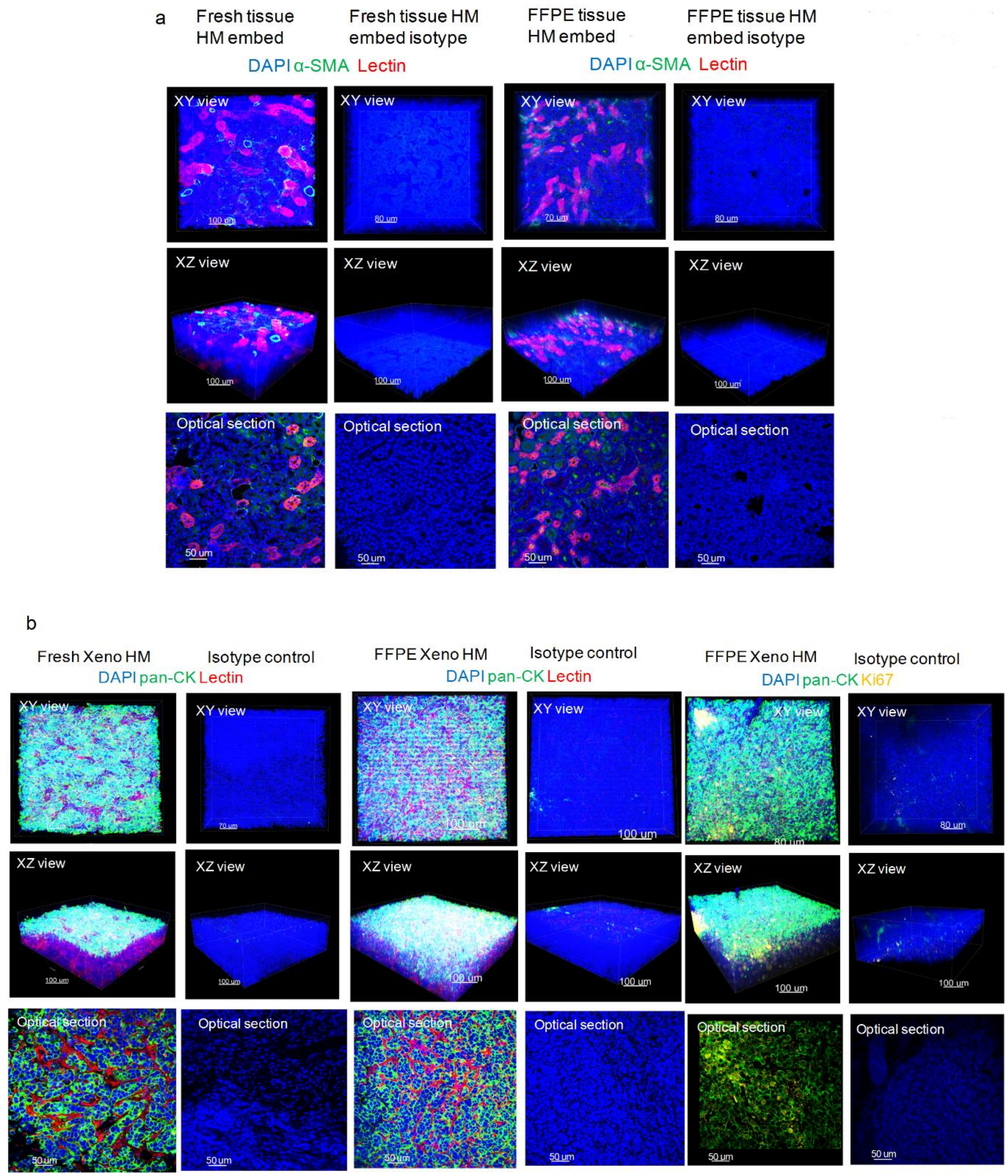

Figure S2: Established the feasibility of processing and immunostaining converted FFPE to HM-embedded mouse kidneys. (a) Fresh mouse kidney tissue (200µm) embedded directly into an A4B4P4 HM solution (1st and 2nd columns) and mouse kidney tissues from FFPE blocks were deparaffinized and re-embedded into an A4B4P4 HM (3rd and 4th columns) were lipid cleared and immunostained with DAPI (blue), α-SMA (green) and lectin (red). 3D volumetric images were shown with XY axes view (top), XZ axes view (middle) and a representative 2D optical section view (bottom) from the respective tissue, 25x. (b) Fresh mouse MCF7 xenograft model tumor tissue (200 µm) were embedded directly into an A4B4P4 HM solution (1st and 2nd columns), and deparaffinized MCF7 xenograft tumor from FFPE blocks that were re-embedded into an A4B4P4 HM (3rd – 6th columns). The tumor tissue was immunostained with antibodies against pan-CK (green) and lectin (red) as shown in columns 1 - 4 (25x). The HM tissue from the FFPE blocks were also stained with nuclei protein marker Ki67 (yellow) to confirm the integrity of nuclei protein (5th and 6th columns) (25x).

SUPPLEMENTAL VIDEO LEGENDS

Video S1: A 3D rendering and 2D slice playthrough of immunostained adjacent normal breast cancer tissue to tumor #7 (A4B4P4). 25x, Blue: DAPI, Green: pan-CK, Yellow: Ki67, Red: CD3.

Video S2: A 3D rendering and 2D slice playthrough of immunostained breast cancer tissue tumor #7 (A4B4P4). 25x, Blue: DAPI, Green: pan-CK, Yellow: Ki67, Red: CD3.

Video S3: A 3D rendering and 2D slice playthrough of immunostaind breast cancer tissue tumor #8 (A4B4P4). 25x, Blue: DAPI, Green: pan-CK, Yellow: Ki67, Red: CD3.

Video S4: A 3D rendering and 2D slice playthrough of the FFPE to HM converted tissue block, breast cancer tissue tumor #7 (A4B4P0), that was then subsequently immunostained. 25x, Blue: DAPI, Green: pan-CK, Yellow: Ki67, Red: CD3.
